# Functionalized Wood Veneers as Vibration Sensors: Exploring Wood Piezoelectricity and Hierarchical Structure Effects

**DOI:** 10.1021/acsnano.2c04668

**Published:** 2022-09-06

**Authors:** Farsa Ram, Jonas Garemark, Yuanyuan Li, Torbjörn Pettersson, Lars A. Berglund

**Affiliations:** ^†^Division of Biocomposites and ^‡^Division of Fibre Technology, ^§^Wallenberg Wood Science Center, Department of Fibre and Polymer Technology, KTH Royal Institute of Technology, Stockholm SE-10044, Sweden

**Keywords:** wood functionalization, nanoengineering, piezoelectric, vibration sensing, sustainable energy technology

## Abstract

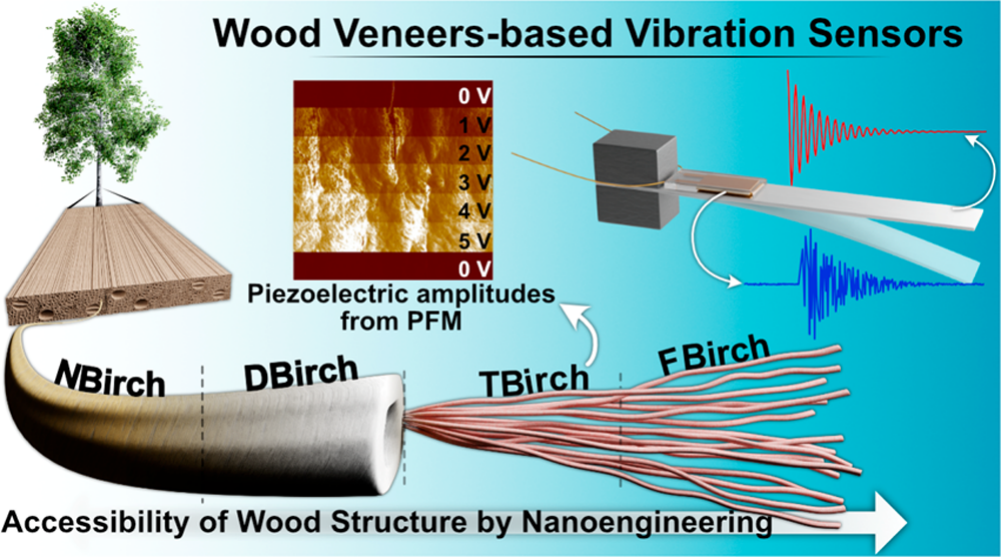

Functional wood materials often rely on active additives due to the weak piezoelectric response of wood itself. Here, we chemically modify wood to form functionalized, eco-friendly wood veneer for self-powered vibration sensors. Only the piezoelectricity of the cellulose microfibrils is used, where the drastic improvement comes only from molecular and nanoscale wood structure tuning. Sequential wood modifications (delignification, oxidation, and model fluorination) are performed, and effects on vibration sensing abilities are investigated. Wood veneer piezoelectricity is characterized by the piezoresponse force microscopy mode in atomic force microscopy. Delignification, oxidation, and model fluorination of wood-based sensors provide output voltages of 11.4, 23.2, and 60 mV by facilitating cellulose microfibril deformation. The vibration sensing ability correlates with improved piezoelectricity and increased cellulose deformation, most likely by large, local cell wall bending. This shows that nanostructural wood materials design can tailor the functional properties of wood devices with potential in sustainable nanotechnology.

## Introduction

Wood veneers are investigated as a green piezoelectric vibration sensor to elucidate the effect of nanostructural design. The anisotropic hierarchical wood structure accessibility was tuned from the macroscale to the nanoscale by mild chemical modifications to improve its piezoelectric properties for vibration sensing. Piezoelectric materials can utilize mechanical energy from vibrations converting it to electricity and act as a self-powered vibration sensor.^1^ An important focus for the choice of materials for vibration sensors is directed toward biobased and sustainable piezoelectric materials.^2^ Wood in this regard is promising due to the advantages of biodegradability, renewability, and sustainability. Cellulose,^3,4^ silk,^5^ and chitin^6^ are among the commonly used biobased piezoelectric materials; however, these materials are usually prepared by energy intensive multistep bottom-up approach, which is less eco-friendly compared to the relatively simpler top-down approach.^7,8^

On the other side, the top-down approach offers easier preparation of functionalized piezoelectric wood composites. The piezoelectricity in wood microfibrils is due to the presence of crystalline native cellulose.^9,10^ The piezoelectric properties vary with wood species and the micro- and macrostructure of wood.^11^ However, low piezoelectric output has restricted the technological applications of wood. Instead, more attention has been paid to cellulose nanofibers (CNF) and cellulose nanocrystals (CNCs) due to their high crystallinity and higher dipole moment.^12,13^ These studies mainly focused on improving the piezoelectric properties by adding functional materials^14^ or enhancing the alignment of the CNFs or CNCs by cold stretching, ice templating, and application of an electrical or magnetic field.^15−17^ Interestingly, wood, which already has naturally aligned fibers/fibrils has seldom been used for such applications.^18^ Recently, Sun et al. reported improved piezoelectric properties by delignification of wood, and prepared balsa wood aerogels are explored for mechanical energy harvesting and demonstrated a few practical applications as a pressure sensor, a wearable sensor, and powered a light-emitting diode.^19,20^ Fukada et al. improved the piezoelectric properties of native wood by tuning the crystal structure using sodium hydroxide, liquid ammonia, or ethylenediamine treatments. The increased piezoelectric properties were attributed to the higher deformability of hydrogen bonds of cellulose II and III compared to cellulose I under applied mechanical stress.^10^ However, not much attention was paid to the possible effect of derivatization of cellulose from these chemical treatments, and prepared wood materials were not explored for practical applications.

Herein, we report nanoengineering of hierarchical anisotropic wood structure to improve the piezoelectric properties for vibration sensing applications. Native wood was sequentially modified by delignification, oxidation, and model fluorination while preserving piezoelectric fibrous cellulose in its native crystalline allomorph. We investigate the effect of these modifications on the molecular and the nanoscale accessibility of wood structures. The functionalized wood veneers were characterized by piezoresponse force microscopy to evaluate their inherent piezoelectricity and were then examined as vibration sensors. Furthermore, effects of functionalization on the vibration sensing ability were investigated for different fiber orientation angles (0° and 45°).

## Results and Discussion

**Figure 1:**
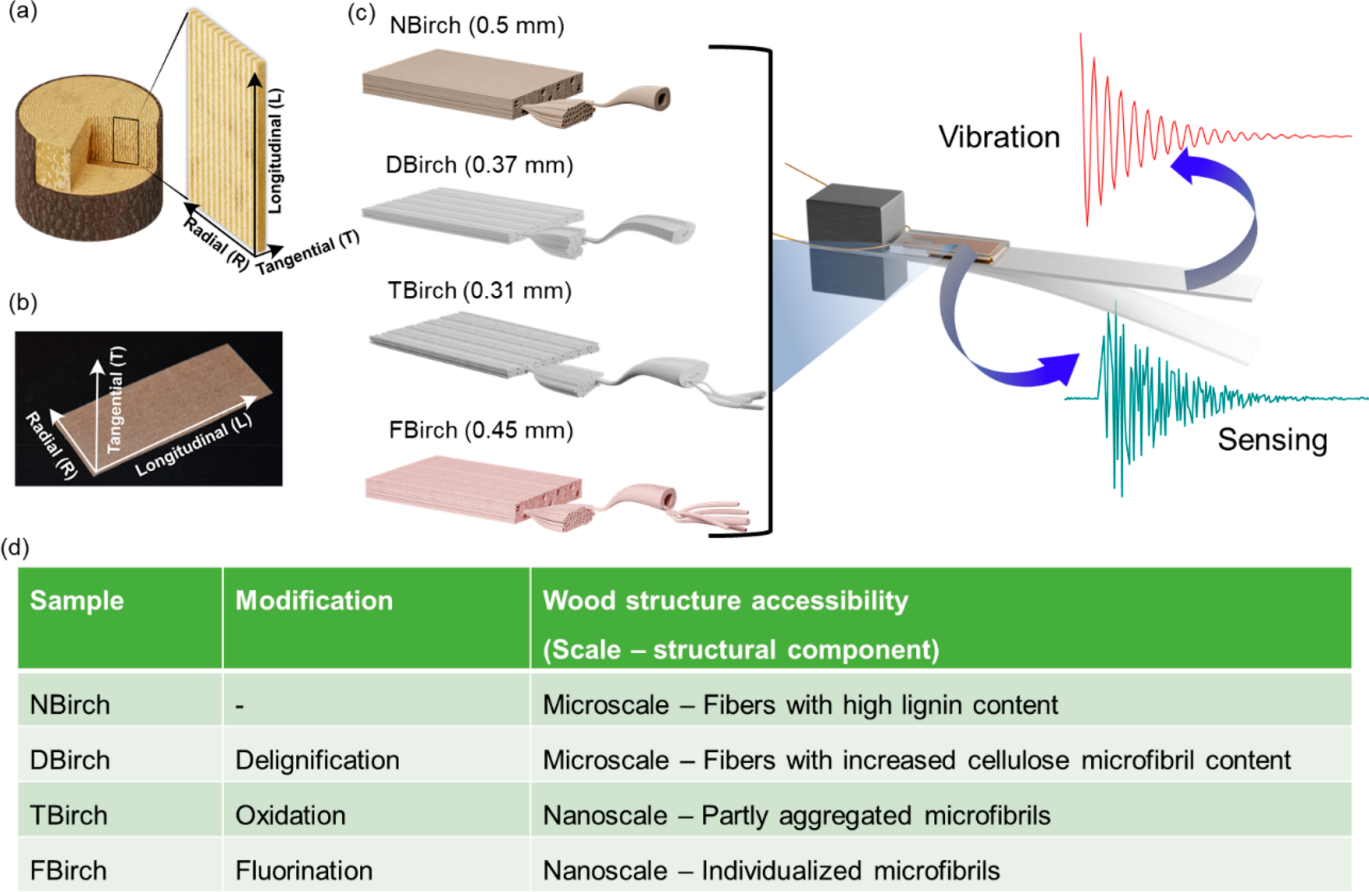
(a) Schematic of wood veneer cut from tree trunk and (b) its digital image showing the longitudinal, radial, and tangential directions. (c) Overview of functionalized wood-based vibration sensors. On the left illustrations show the functionalized wood veneers. The thicknesses of veneers varied after treatments and are mentioned in parentheses. On the right, a sensor fabricated from functionalized wood veneers is mounted on the beam. The simulated signal from vibrations is in red, and sensing is presented in the dark cyan signal as measured voltage. (d) Types of modification and accessibility of the wood microstructures shown in Figure 1c.

Different wood substrates are prepared by wood cell wall functionalization (including surfaces of cellulose microfibrils) and investigated to clarify the details of chemical modification and hierarchical structure effects on vibration sensing. The nature of the hierarchical structure is clarified in Figure 1a–c, where the porosity of wood is apparent as well as the tubular shape of the interconnected wood fiber cells. An important aspect of structural hierarchy is that the wood cell wall is reinforced by axially aligned, high modulus (*E* ≈ 100 GPa) cellulose microfibrils with a diameter of ≈3–4 nm.^21^ The present wood vibration sensing is realized due to the piezoelectric properties of wood, where the mechanical vibrations are converted into electrical signals and measured as an output voltage. An overview of the whole process is illustrated in Figure 1c. Three types of functionalizations are carried out: (a) delignification to improve relative cellulose content and internal cell wall accessibility for further modification, (b) TEMPO-mediated oxidation of delignified wood by oxidation of hydroxyls to carboxyl groups, and (c) fluorination of TEMPO-oxidized wood veneers by reaction of carbonyl groups with trifluoroethylamine. The hypothesis is that cellulose microfibrils in the cell wall can stay individualized at the nanoscale, even after drying at ambient conditions. Ambient drying is otherwise known to lead to cellulose microfibril aggregation.^18^ Because of structural hierarchy, effects can be present on the micro and nanoscale; see table in Figure 1b. Native wood, delignified wood, TEMPO-oxidized wood, and fluorinated wood are labeled as NBirch, DBirch, TBirch, and FBirch, respectively.

**Figure 2:**
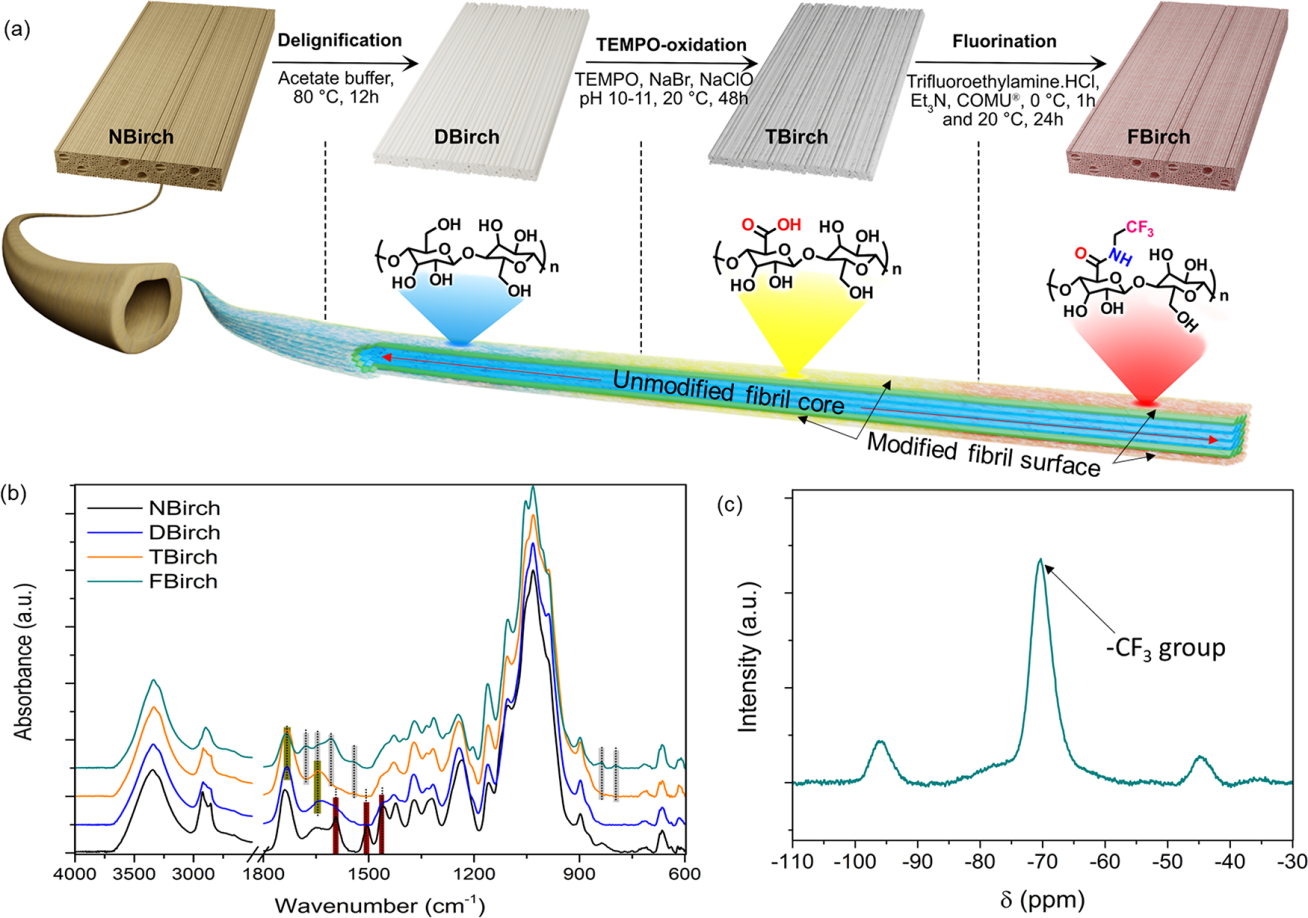
(a) Reaction scheme of chemical modification of birch wood by delignification, TEMPO oxidation, and fluorination and their FTIR spectra (b). (c) Solid-state ^19^F magic angle spinning nuclear magnetic resonance (MAS NMR) spectra of FBirch showing the presence of the −CF_3_ group.

Figure 2a shows a sketch of the different birch wood modifications, which will be described: Native birch was first delignified (see methods section). Delignified birch was further oxidized using TEMPO as a catalyst under alkaline conditions. The reaction only occurs at the cellulose microfibril surfaces, cellulose is accessible, with no modification inside the ≈3–4 nm diameter elementary cellulose fibrils. Under the present oxidative conditions, primarily −OH groups of C6 carbons at cellulose fibril surfaces are oxidized to carboxylic acid groups, although hemicelluloses will also be functionalized. The charge density of TEMPO-oxidized birch (TBirch) was ≈450 μmol/g. Never-dried TBirch samples were then subjected to solvent exchange from ethanol to acetonitrile to perform an amide coupling reaction with trifluoroethylamine. This reaction was assisted by a uronium-based green amide coupling reagent COMU (in accordance with the 12th principle of green chemistry). Note that also the fluorination was limited to the fibril surfaces in the cell wall and carried out through the thickness of wood veneer as supported by fluorine mapping on the cross-sectional image of FBirch (Figure S1). Finally, functionalized wood veneers were dried under ambient conditions and used for further analyses. Figure 2b shows the Fourier transformed infrared (FTIR) spectroscopy data of native and functionalized birch wood veneers. The peaks corresponding to lignin at 1462, 1505, and 1590 cm^–1^ disappeared or intensities were decreased.^22^ Further, with TEMPO oxidation the intensity of carbonyl peaks near 1645 and 1733 cm^–1^ increased, in support of successful functionalization. In FBirch, the intensity of carbonyl peaks near 1733 and 1645 cm^–1^ decreased, and two new peaks appeared at 1681 and 1607 cm^–1^ due to amide bond formation. Additionally, the appearance of two new small peaks near 795 and 840 cm^–1^ corresponds to the −CH_2_–CF_3_ in the FBirch. Fluorination was further confirmed by solid-state ^19^F MAS NMR spectra of a powdered sample of FBirch. In Figure 2c, the presence of a peak near δ = −70.3 ppm corresponds to −CF_3_ groups confirming the fluorination in FBirch.^23^

**Figure 3:**
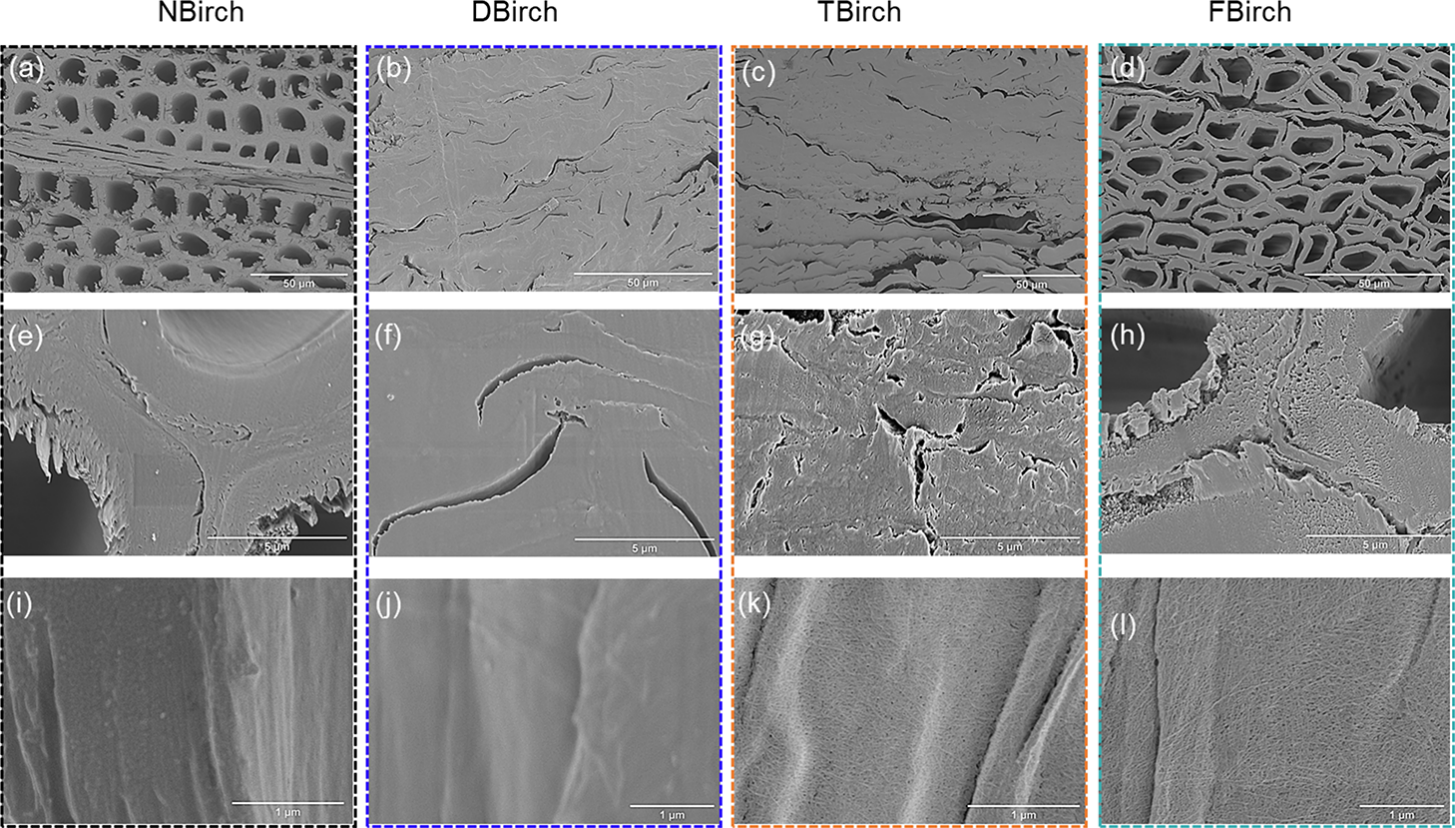
Cross-sectional FE-SEM micrographs of NBirch, DBirch, TBirch, and FBirch. (a), (b), (c), and (d) are overviews of samples; no fibrillation is observed in zoom-in images of the middle lamella and interfiber boundaries for (e) NBirch and (f) DBirch, while fibrils are apparent in (g) TBirch and (h) FBirch. The surface morphology of fibers (L-R surface) shows no individual fibrils in (i) NBirch and (j) DBirch, while (k) and (l) show individual fibrils in TBirch and FBirch.

The physical wood substrate structure is critical for mechanical performance as well as for device functionality. Field emission scanning electron microscopy (FE-SEM) is used to analyze the cross sections and longitudinal sections of the wood veneers, and their micrographs are shown in Figure 3. The cross-sectional structure shows the original fiber structure of native birch (Figure 3a), the spontaneously densified structure in DBirch and TBirch from drying (Figure 3b,c), and FBirch, where wood structure resembles native wood (Figure 3d). Subsequent panels e–h show zoomed-in images of interfiber cell boundaries. DBirch and TBirch wood structures were densified due to capillary forces developing during ambient drying (Figure 3f,g). Interestingly, fluorination prevents structural collapse and densification under ambient drying since the surface energy is reduced. Interfiber boundaries show fibrillar cell walls due to fluorination; see Figure 3h. Figure 3i–l and Figure S2 show fiber cell surfaces (longitudinal sections) in wood veneers. The fibrillar structure in TBirch and FBirch is also apparent in Figure 3k,l. Note that the cell wall is accessible at molecular scale (nanoporous) (Figure S3).^18^ In NBirch and DBirch, fibrils are embedded in the retained lignin–hemicellulose matrix, and fibrils are not observable in SEM images (Figure 3i,j).

**Figure 4:**
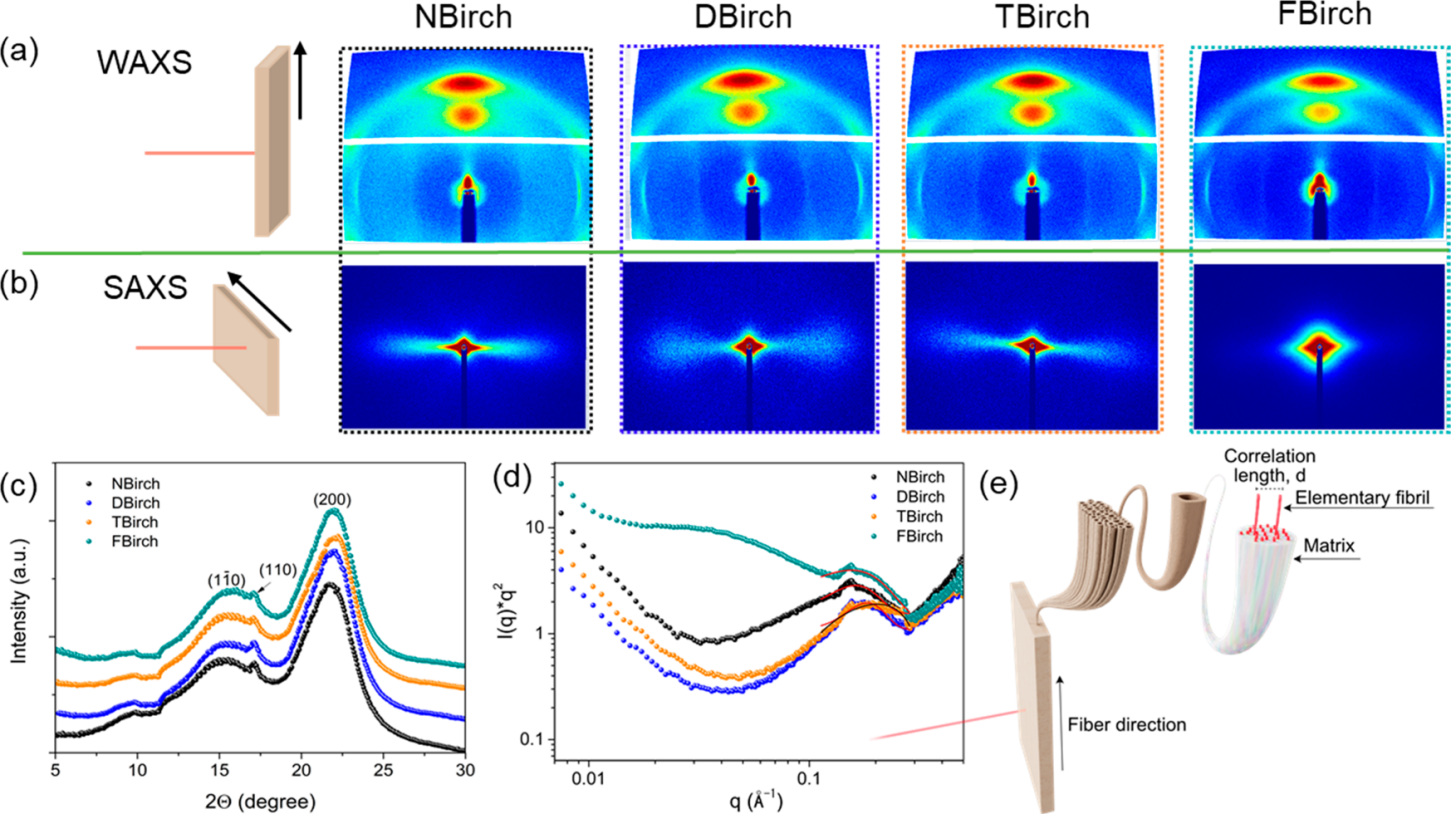
(a) WAXS and (b) SAXS 2-D scattering profile of birch wood samples (black arrows direction on the top sketch represents the direction of fiber orientation) shows the oriented fibrils, where 2-D SAXS scattering is distinguishable. Red lines indicate the beam. 1-D profile of (c) WAXS shows that surface modification has not altered crystalline nature (cellulose I allomorph). (d) *I*(*q*)*q^*2*^ vs *q* from 1-D SAXS data of wood samples. (e) Illustration of the hierarchical structure of wood from veneer to wood tissue to fiber cells to elementary fibrils. Correlation length between elementary fibrils embedded in “matrix” (hemicellulose and residual lignin) is derived from SAXS measurements.

Supramolecular and nanoscale structures of the wood samples were investigated by wide and small-angle X-ray scattering (WAXS and SAXS); see Figure 4. Two dimensional (2-D) scattering images from WAXS and SAXS show preserved anisotropic structure after functionalization, Figure 4a,b. The elliptical scattering patterns confirm molecular orientation in nanoscale fibrils.^24^ The so-called “top-down” approach used here means that the native, pre-existing cellulose microfibril orientation and nanoscale dispersion in wood are utilized, rather than first disintegrating the plant cell wall to extract nanocellulose fibrils (CNF) and then trying to disperse and orient them in new materials.^25^ The wood fiber directions in wood veneers are illustrated at the side of the respective 2-D scattering images. The 1-D WAXS data (Figure 4c) suggests that all wood veneers consist of cellulose in the cellulose I crystalline allomorph; chemical modifications have not changed the crystalline allomorph of wood cellulose microfibrils. Furthermore, the crystallinity index in DBirch, TBirch, and FBirch was increased to 53.6, 52.5, and 55.1% from 46.2% of NBirch (Table S1). We then probed nanostructural changes in wood veneers from functionalization, using SAXS. This is a very powerful technique to determine the structure of the wood cell wall, which is considered as a nanomaterial dominated by elementary cellulose nanoscale fibrils oriented in parallel. The *I*(*q*)*q^*2*^ vs *q* plot derived from the 1-D profile of SAXS data (Figure S4) is represented in Figure 4d. FBirch has dramatically different 2-D scattering compared to other samples due to increased interfibrillar correlation length (*d*), which is illustrated in Figure 4e.^26^ In NBirch, fibrils are densely packed in a hemicellulose–lignin matrix and have an interfibrillar correlation length of ≈3.88 nm (Table S2). Lignin removal followed by ambient drying results in cell wall collapse (due to capillary forces) so that the interfibrillar distance in DBirch is reduced (*d* ≈ 3.49 nm). The interfibrillar distance was further reduced to ≈3.09 nm in TBirch, probably due to stronger densification related to the carboxyl groups from TEMPO oxidation.^18^ When these carboxyl groups were modified with trifluoroethylamine (FBirch), the lowering of the surface energy resulted in negligible collapse and densification, and the elementary fibril correlation distance from SAXS was ≈4.07 nm. This observation also means that there is nanoscale porosity in the FBirch cell wall.

**Figure 5:**
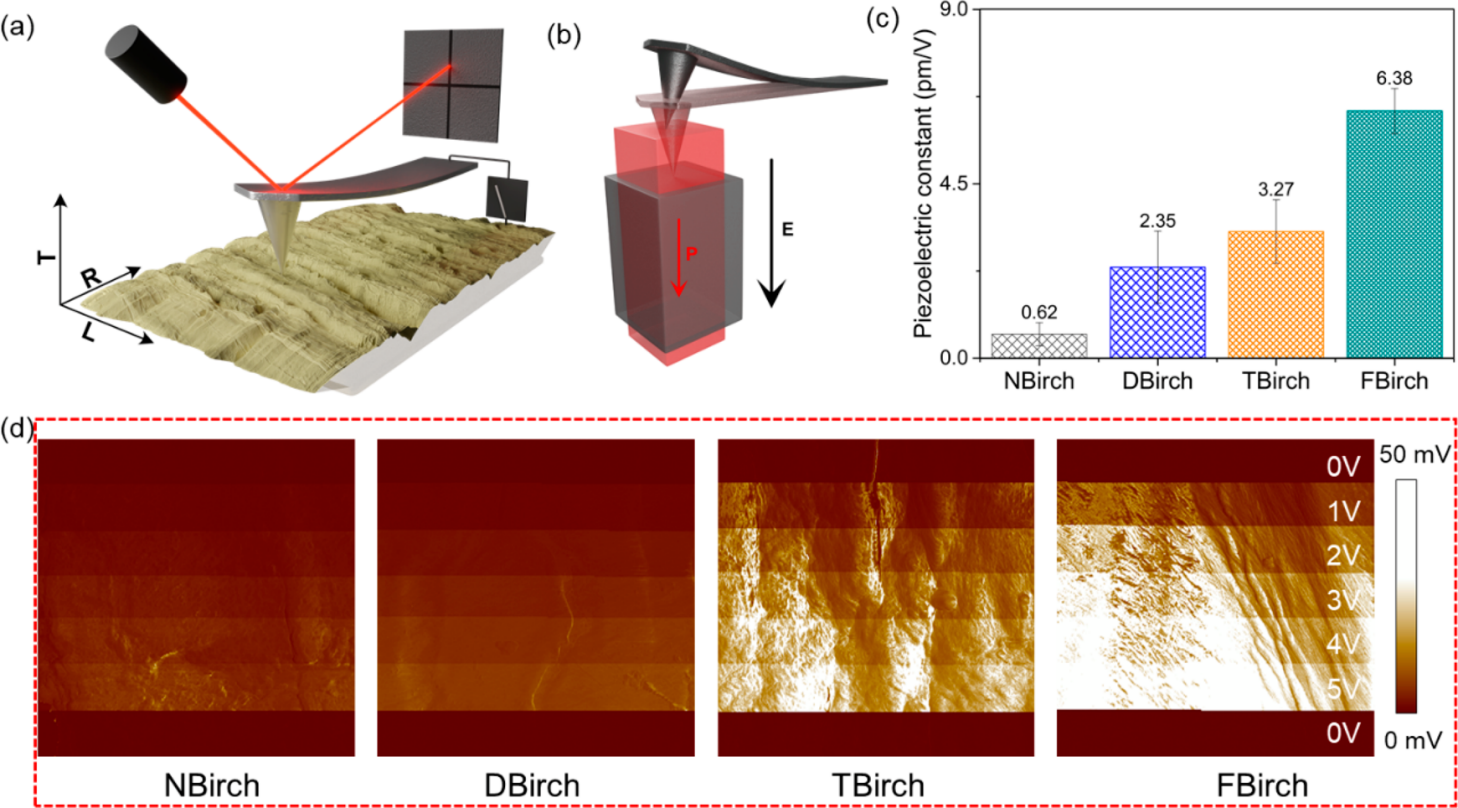
(a) Illustration of piezoresponse force microscopy (PFM) measurements on wood substrates showing the tip engagement on the L-R surface (approached from the top or normal to the L-R plane). (b) Schematic of working principle of PFM. When the positive bias is applied, the alignment of the electric field (*E*) and polarization (*P*) orientation results in an expansion of the piezo material under the tip, giving a positive deflection, which is then measured by the photodiode and retrieved as the amplitude. (c) The effective piezoelectric constant (*d*_eff_) of wood samples and (d) variation of amplitude with applied bias show the piezoelectric behavior. The PFM scan size is 5 μm × 5 μm; legends and *z*-scale apply to all the images.

The piezoelectric properties of the functionalized wood veneers were then investigated. The piezoelectric performance of a material is defined by the piezoelectric modulus or constants, which are defined as the charges generated per unit force (pC/N) or the extent of actuation per unit applied electric field (pm/V). Wood (cellulose crystal with *C*_*2*_ symmetry) may exhibit eight piezoelectric constants, namely, *d*_14_, *d*_15_, *d*_24_, *d*_25_, *d*_31_, *d*_32_, *d*_33_, *d*_36_. Among them, *d*_14_ and *d*_25_;^10^*d*_31_, *d*_32_, and *d*_36_;^27^ and *d*_33_(28) have been measured experimentally in various wood species. Piezoresponse force microscopy (PFM) can be used to characterize the piezoelectricity. Figure 5 panels a and b illustrate PFM in atomic force microscopy (AFM). Upon applying a positive bias, the alignment of the applied electric field (*E*) and the resultant polarization (*P*) of the piezoelectric material result in mechanical deformation (positive deflection), which is sensed by the photodetector. The mechanical deformation/deflection retrieved as the amplitude is a function of applied bias. All the wood materials responded to the applied bias, and their amplitude increased as the applied bias was increased from 1 to 5 V. The piezoelectric constant (*d*_33_, applied load, and polarization in the *z*-axis) was calculated as the effective piezoelectric constant (*d*_eff_) shown in Figure 5c, which was deduced from the piezoelectric amplitudes (refer to the Experimental Section). The variation of amplitude with the applied bias (Figure 5d) confirms the inherent piezoelectric nature of the wood materials. The origin of piezoelectricity is attributed to the net dipole moment arising from noncentrosymmetrically arranged hydroxyl groups^29^ and inter- and intralayer hydrogen bonds between these hydroxyl groups in crystalline cellulose of wood microfibrils.^30^ Note that the piezoelectric constants characterize the inherent piezoelectric nature of wood veneers and vary with the micro- and macrostructure of wood.^11^ With increased cellulose content and increased “separation” of individual cellulose microfibrils, i.e., larger correlation distance, the samples correlate with their enhanced piezoelectric properties (*d*_eff_) in the order NBirch (0.62 pm/V) < DBirch (2.35 pm/V) < TBirch (3.27 pm/V) < FBirch (6.38 pm/V). The piezoelectric constant values of functionalized wood veneers were comparable to or higher than other natural polymeric materials, e.g., uniaxially oriented silk film (1.5 pC/N),^5^ chitin (0.2–1.5 pC/N),^32^ and CNF film (5.7 ± 1.2 pC/N).^15^ The imparted functionalities may contribute toward the improved piezoelectric output. However, considering the low functionalization densities, the contribution should be minimal.^10,33^ This is only one piezoelectric constant among the eight possible ones for wood, so one may speculate that in a porous wood substrate the contribution from the shear piezoelectric constant may be important.

**Figure 6:**
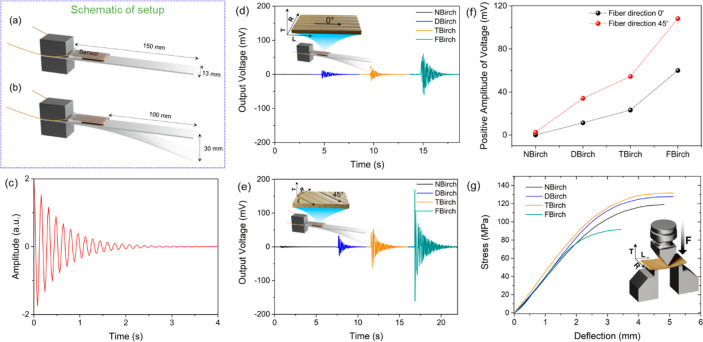
Schematic setup for generating decaying vibration using a cantilever beam, (a) a sensor mounted on a cantilever beam, (b) deflection of a cantilever beam to produce vibration, and (c) a simulated decaying vibration signal produced by deflection of the cantilever beam. Piezoelectric output voltage generated by sensing of vibrations from the beam, (d) when fiber orientation is parallel to the beam length (fiber direction 0°) and (e) when fiber orientation is 45° to the beam length (fiber direction 45°), and comparison of the positive amplitude of voltage output when the device has fiber length directions at 0 and 45°. (g) Stress–deflection curves of the wood veneers from a three-point bending test. The inset image shows that the load was applied perpendicular to the L-R surface.

Figure 6 shows the setup for vibration generation, the vibration sensing ability of the sensors from functionalized wood veneers, and the mechanical properties of functionalized wood veneers. Vibrations were generated by deflecting the free end of a cantilever beam while one end was fixed as illustrated in Figure 6a,b. An example of such vibrations is simulated and plotted in Figure 6c. When the wood fibers are oriented along the long axis of the sensor and the beam, the output voltages produced by vibration sensing are plotted in Figure 6d. The active vibration frequencies were deduced by fast Fourier transformation (FFT) of the voltage signal and found to be between 2 and 20 Hz (with a dominant frequency close to the natural frequency of the beam ≈18 Hz); see Figure S5. NBirch is inactive toward vibration sensing, and there was no active vibration frequency (Figure S5) due to low effective cellulose content. Apparently, the lignin phase (relative content ≈20%) in native wood is reducing sensitivity. With increased cellulose content (from ≈55 to 69.5%, Table S1) after delignification, DBirch with a relative lignin content of ≈3.4% resulted in an output voltage of 11.4 mV from induced vibration, hence showing the vibration sensing. The output voltage was further increased for TBirch (23.2 mV). This correlates with increased individualization of elementary cellulose fibrils of ≈3–4 nm diameter and increased polarity from the added carboxylic acid groups. However, the effect of the latter will be lesser due to the low density of carboxyl groups (≈450 μmol/g).^10^ Moreover, individualization of elementary cellulose fibrils was not effectively utilized in TBirch since the wood cell wall collapsed during ambient drying (evident from reduced porosity and thickness and increased density, Table 1). For FBirch, the addition of −NH–CH_2_–CF_3_ prevented fibril aggregation during drying (Figure 3, FE-SEM images and Figure 4, SAXS data), and the cellulose fibrils are discrete at the nanoscale with some cell wall porosity after drying (Table 1). Hence, FBirch provides the highest output voltage of 60 mV compared with the other wood-based sensors. Although fluorination of cellulose surfaces is used, it is a model experiment, here shown to be efficient in separating cellulose microfibrils for improved sensing performance. Furthermore, the performance of nonfluorinated sensors (TBirch) is also high. The sensors also show similar properties even after prolonged storage of 5–6 months (Figure S6), suggesting their durability in practical conditions.

**Table 1:** Density, Porosity, and Mechanical Properties of Functionalized Wood Veneers

sample	thickness (mm)	bulk density (g/cm^3^)	porosity (%)	flexural strength (MPa)	flexural modulus (GPa)
NBirch	0.5	0.52	63.16	117 ± 5.2	12.6 ± 0.7
DBirch	0.37	0.68	52.97	121 ± 5.9	15.6 ± 0.4
TBirch	0.31	0.73	50.22	135 ± 7.7	19.4 ± 0.3
FBirch	0.45	0.44	68.98	86.2 ± 5.7	12.1 ± 1.3

When the long axis of fibers is oriented at 45° off-axis angle to the long axis of the beam, the vibration sensing efficiency is further enhanced with even higher output voltages (DBirch (34.1), TBirch (54.5), FBirch (108.2)), as shown in Figure 6e, and the positive amplitudes of voltages of the wood sensors are plotted in Figure 6f. The increased vibration sensitivity in 45° oriented fibers is attributed to the increased local small-scale shear between fibers and between fibrils compared to 0° oriented fibers. Our findings are in agreement with results from Nakai et al., where at the 45° fiber orientation a higher piezoelectric output was obtained.^34^ Hence, for 45° off-axis orientation of the wood fibers in the sensor, the NBirch (2.6 mV) was also able to sense the vibrations. Active vibration frequencies were similar to functionalized wood veneer sensors (Figure S7). Within the functionalized wood veneers, the increased cell wall porosity in FBirch probably contributed to higher local shear strain, and the highest output voltage of the study was obtained, providing improved sensor function. The output voltages from these sensors are higher than previous wood or natural polymer-based materials, e.g., 19.1 mV (45° oriented spruce wood sample of size 0.5 × 1 × 6 cm^3^)^35^ and 3 mV (chitosan–formate film).^36^ These vibration sensors have potential applications in structural health monitoring of infrastructures (e.g., bridges) and appliances. Every running machine and infrastructure produces vibrations with a certain frequency. The change in vibration frequency can indicate the malfunctioning or the presence of external stimuli, which can be resolved before actual failure.

During vibration sensing, vibrations may damage the microstructure and reduce the structural integrity of the sensing material. It is important to utilize chemically modified surface functionalities for enhanced piezoelectric output of wood while retaining mechanical properties. The mechanical properties were measured by three-point bending which induces tensile (lower specimen region) and compression (upper specimen region) stress. All specimens showed linear behavior until a deflection of about 1.5 mm, which indicates the maximum deformation prior to microstructural damage. NBirch and DBirch showed flexural strengths of 117 ± 5.2 and 121 ± 5.9 MPa, respectively. The flexural modulus was increased from NBirch (12.6 ± 0.65 GPa) to DBirch (15.6 ± 0.43 GPa) as listed in Table 1. The main reason for this increase is that DBirch is collapsed with large reduction in porosity; see Figure 3 and Table 1. TBirch, which also collapsed, showed a flexural strength of 135 ± 7.7 MPa and a flexural modulus of 19.4 ± 0.32 GPa due to even higher densification, evident from reduced porosity and thickness during ambient drying (Tables 1). The flexural strength of FBirch was 86.2 ± 5.7 MPa with a flexural modulus of 12.1 ± 1.3 GPa, which is the lowest among the functionalized wood veneers and even lower than NBirch since there is no collapse during drying and the porosity is high (porosity of ≈69%). The mechanical properties of functionalized wood substrates are substantial for designing vibration sensors, and mechanical properties are higher for the functionalized wood compared to commercial synthetic piezoelectric polymers, e.g., poly(vinylidene fluoride).^37^ Additionally, due to the tuned surface polarity, the TBirch–FBirch combination (oxidized–fluorinated) was utilized as tribo-positive and tribo-negative materials with tuned surface chemistry for fabrication of a triboelectric nanogenerator. This triboelectric nanogenerator resulted in the output voltage of ≈6 V over 5000 cycles (Figure S8 and associated discussion).

## Conclusions

A green materials concept for vibration sensing is presented using functionalized wood veneer with enhanced piezoelectricity by tuning the wood structure at the molecular and nanoscale levels. The vibration sensing ability was in the order of DBirch < TBirch < FBirch (delignification < oxidation < model fluorination) correlated with increased nanoscale individualization of elementary cellulose fibrils in the wood cell wall. This translates into increased local deformation of individual cellulose fibrils during sensing. The high cell wall porosity resulting from delignification resulted in increased nanoscale interfibril distance in FBirch (supported by porosity data and SAXS analysis), and higher local deformation for improved vibration sensing ability. The vibration sensing ability further improved in sensors with fibers oriented at 45°, where deformation under shear stress is increased. As the cell wall is subjected to bending, the local cellulose microfibril deformation can be very large (with increased piezoelectric response), related to the hierarchical wood structure. In conclusion, nanoengineering of a hierarchical wood structure was performed by mild chemical treatments, and this top-down strategy is utilizing the orientation of cellulose fibrils in the wood cell wall, with potential for nanotechnology applications of sustainable wood materials.

## Experimental Section

### Materials

2,2,6,6-Tetramethyl-1-piperidinyloxy (TEMPO), sodium bromide (NaBr), 1-cyano-2-ethoxy-2-oxoethylidenaminooxy)dimethylaminomorpholinocarbenium hexafluorophosphate (COMU), and trifluoroethylamine hydrochloride (TFEA·HCl) were purchased from Sigma-Aldrich, Sweden, and used as received. Sodium hydroxide (NaOH), sodium hypochlorite (NaOCl), sodium acetate trihydrate, sodium chlorite, ethanol, and acetonitrile were purchased from VWR, Sweden. Deionized water was used from a laboratory setup. The birch wood (*Betula pendula*) was purchased from Fredricsons Trä, Sweden.

### Delignification of Wood

Lignin was removed by treating the wood veneers (50 mm × 20 mm × 0.5 mm in longitudinal × radial × tangential directions) in 1 wt % NaClO_2_ solution in sodium acetate–acetic acid buffer (pH ≈4.6) at 80 °C for 12 h.

### TEMPO Oxidation of Wood

A 0.2 g sample of TEMPO and 1 g of NaBr were added to deionized (DI) water and stirred for 5 min at room temperature. Then the delignified wood veneers (5 × 2 cm^2^) were transferred to this solution and 10 mL of 14 wt % NaClO was added. The pH of the solution was adjusted to 10–11 by 3 N NaOH, and the reaction was kept for 48 h at room temperature. After 48 h, the wood veneers were washed with DI water and the carboxylates were converted into free acid form by two more washing cycles with 0.1 N HCl. Excess HCl was washed-off until the conductivity of the supernatant was below 5 μS/cm. The carboxyl content was determined by titrating with NaOH using a Metrohm 856 automated conductometer. The carboxyl content was calculated based on the NaOH consumption, which was found to be ≈450 μmol/g.

### Fluorination of Wood

Never-dried oxidized wood veneers (TBirch) were subjected to sequential solvent exchange from water to ethanol and then to acetonitrile. In a separate bath 2.7 mL of triethylamine was added to 200 mL of acetonitrile at 0 °C followed by stirring for 2 min. Additionally, 1.3 g of TFEA·.HCl and 1.13 g of COMU were dissolved in ethanol and acetonitrile, respectively, at 0 °C. All the reactants were mixed in an acetonitrile bath by stirring for 2 min, which turned the solution yellow. Finally, the carboxylated wood veneers were transferred to the bath, and the reaction was processed without stirring to avoid disintegration of the wood veneers. After 1 h, the ice bath was removed to bring the reaction to room temperature and further kept for 24 h. After 24 h, wood veneers were washed with acetonitrile several times until the supernatant was a clear solution and dried at room temperature in a fume hood. An overall process of materials preparation is illustrated in Scheme S1.

### Material Characterization

The morphology of wood samples was analyzed by FE-SEM using a Hitachi S-4800, Japan. Cross sections were obtained by freeze-fracturing in liquid nitrogen followed by drying in a critical point dryer. Energy dispersive X-ray spectroscopy (EDS) equipped on the FE-SEM (Oxford Instruments, X-MAX N 80, UK) was used to evaluate the presence of elements. The functionalization was characterized by FTIR using a PerkinElmer spectrum 100 FT-IR equipped with an MKII Golden Gate, single-reflection accessory unit with a diamond ATR crystal (Graseby Specac Ltd., UK). The spectra were recorded at room temperature in the range 4000–600 cm^–1^. ^19^F NMR MAS spectrum of FBirch was recorded using a Bruker Avance HD 500 MHz spectrometer at the resonance frequency of 470 MHz. The spectrum was recorded at the spinning speed of 12 kHz using a single excitation 90° pulse of 5 μs. The number of scans was 512 with relaxation delay 3 s. The chemical shift was referenced using C_6_H_5_F as the external standard. WAXS and SAXS characterization were performed to check the crystallinity and fiber/fibrillar orientation using a SAXSpoint 2.0 WAXS/WAXS/GISAXS instrument, Anton Paar. The instrument is equipped with an X-ray source of supernova copper, a wavelength of 1.541 Å, ASTIX optics, beam collimation, and an Eiger R 1 M Horizontal detector. The 2-D scattering was converted into 1-D data using acquisition software SAXSanalysis, and all the data were background subtracted before analysis. A three-point bending test was performed using a universal testing machine (Instron 5566, UK) equipped with a 500 N load cell at a strain rate of 10%/min, and the tests were carried out in an environment-controlled room at a temperature of 22 °C and relative humidity of 50%. The flexural modulus was calculated using the following formula:
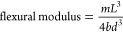
where *m* stiffness is calculated from the slope of force vs the deflection plot in the linear region and *L*, *b*, and *d* are the length, width, and thickness of the wood veneers, respectively.

The solid density of the wood veneers was obtained by pycnometry, and the bulk densities of the wood veneers were calculated by drying the samples at 105 °C until a constant weight was obtained. The porosity was calculated using the following equation:
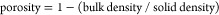


### Piezoelectric Properties

The piezoelectricity of the chemically modified wood veneers was tested using piezoresponse force microscopy on an atomic force microscope (Multimode, 8 equipped nasoscope V controller). All the measurements were carried out in contact mode using a Pt-coated conductive tip HQ:XSC11/Pt (μmash) having a spring constant of 0.178 N/m (normal spring constant was determined by the built-in thermal tune method^38^ using deflection sensitivity measured on fused silica prior spring constant determination). The piezoelectric amplitudes were recorded as a function of applied bias. To calculate the piezoelectric constant (*d*_33_), a standard piezoelectric periodically poled lithium niobite (PPLN, Asylum research, Germany) with a nominal piezoelectric coefficient of 7.5 pm/V was utilized to deduce the calibration parameter from the equation below:^17^
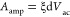
where *A*_amp_ is the piezoelectric amplitude, ξ is the calibration parameter, *V*_ac_ is the applied bias, and *d*_33_ is the piezoelectric constant. The piezoelectric constant (*d*_33_) for all wood materials was calculated from a linear fit of the piezoelectric amplitude vs the applied bias and normalized using the calibration parameter, and it is termed the effective piezoelectric constant (*d*_eff_).

### Vibration Sensing

For vibration sensing, the devices were fabricated from wood veneers by cutting in 1.3 × 2.5 cm^2^ sizes with the desired fiber direction and coating with a 20 nm gold layer using the Eurovac UHV deposition system, followed by attaching a copper tape, leaving the side corner to avoid a short circuit. A set of wires were soldered on the bottom and top electrodes to make the connection for the measurements. Finally, the device was encapsulated in polypropylene tapes to isolate it from environmental and other contributions. The devices were mounted toward the fixed end of a cantilever beam. The decaying vibrations were produced by deflecting the beam by 3 cm and the output response was measured using Keithley’s DMM 7510.

## Supporting Information Available

Crystallinity index (CI), solid densities, and carbohydrate analysis of all wood veneers; SEM images of TBirch, FBirch, and elemental mapping of FBirch; 1-D SAXS data and table for interfibrillar distance; vibration frequency calculation, stability of vibration sensing signals, FFT analysis of voltage signals of vibration sensors with fiber angles at the 0 and 45°; triboelectric mechanical energy harvesting; and schematic for the preparation of functionalized wood veneers (PDF)
