# Supplementary material for: Functionalized Wood Veneers as Vibration Sensors: Exploring Wood Piezoelectricity and Hierarchical Structure Effects
Source: ACS Nano. 2022 Sep 6;16(10):15805–13. doi: 10.1021/acsnano.2c04668 (PMC9620403; doi:10.1021/acsnano.2c04668)
Supplement: Supplementary file 1 — nn2c04668_si_001.pdf [file nn2c04668_si_001.pdf]

Functionalized Wood Veneers as Vibration Sensors –

Exploring Wood Piezoelectricity and Hierarchical Structure Effects

Farsa Ram,[1,3]** Jonas Garemark,* [1,3] *Yuanyuan Li,* [1,3] Torbjörn Pettersson,[2,3] *Lars A. Berglund*[1,3]

[1]Division of Biocomposites, and [2]Division of Fibre Technology; [3]Wallenberg Wood Science Center, Department of Fibre and Polymer Technology; KTH Royal Institute of Technology, Stockholm SE-10044, Sweden.

Corresponding author: farsa@kth.se*

SEM analysis:

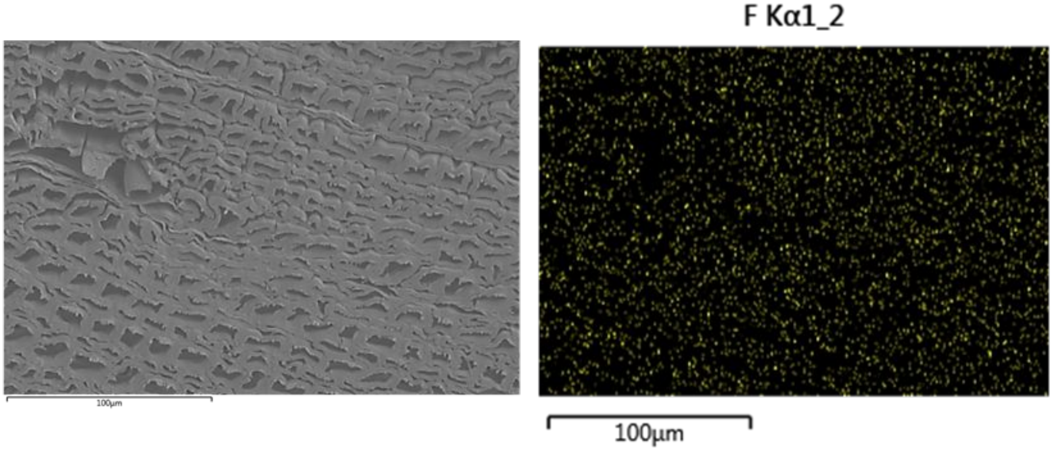

Figure S1: Cross-sectional SEM image (left) and corresponding fluorine distribution in energy dispersive x-ray spectroscopy (EDS) image of FBirch, showing fluorination throughout the thickness of wood veneer.

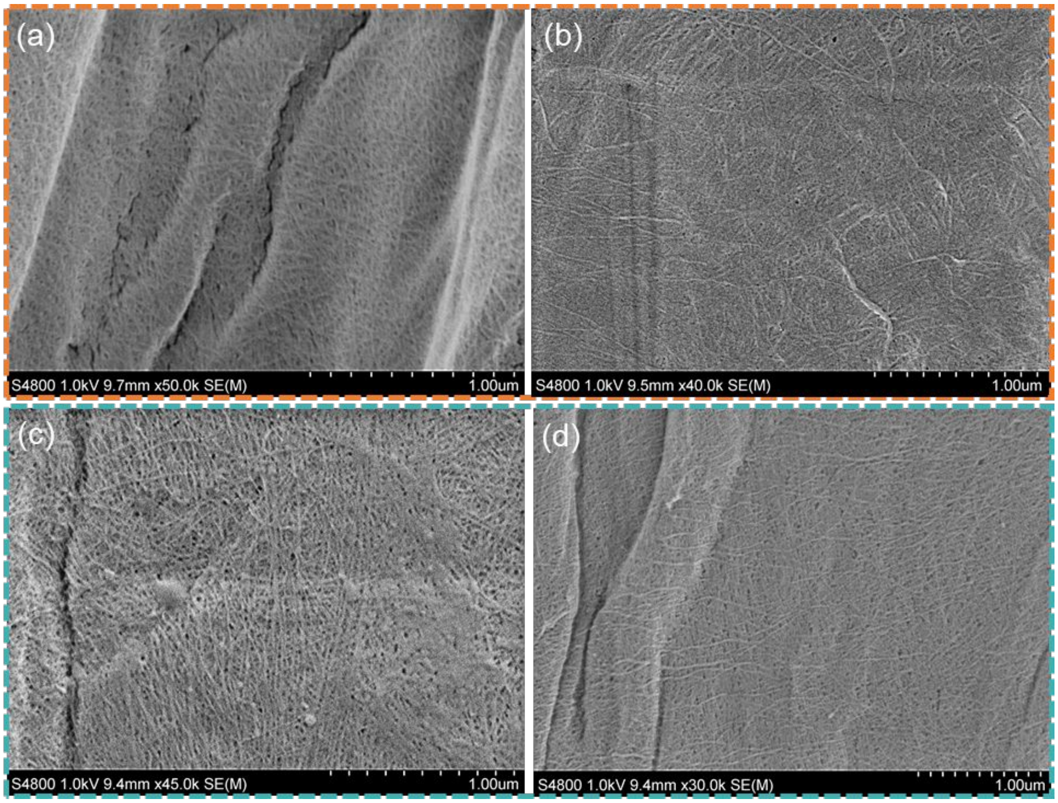

Figure S2: Wood microfibrils in TBrich (a,b) and in FBirch (c,d).

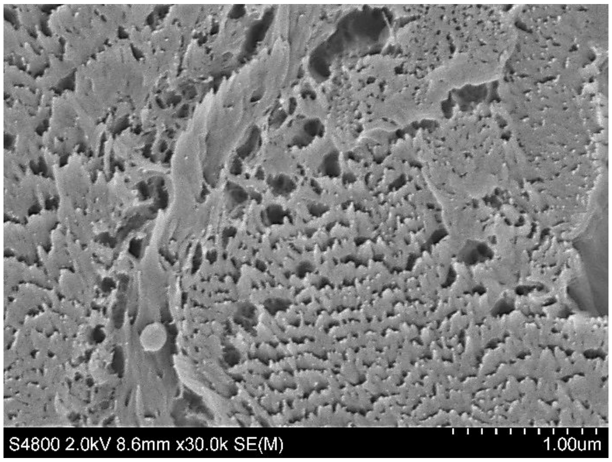

Figure S3: Exposed fibrils in cross section of FBirch fiber wall.

Carbohydrate and crystallinity analysis**:** The carbohydrate analysis was performed in three steps, a) Grinding in Wiley mill, b) hydrolysis by sulfuric acid and c) analysis using a Dionex ICS-3000 ion chromatography system (Thermo Fisher Scientific Inc., USA). The TAPPI T222 om-2 method was utilized for the determination of the lignin content. The crystallinity index (*CI*) of wood veneers was calculated using method by Segal et al.[S1], which employs a simple calculation based on the intensity of the (200) peak (I_{200}) and the indicator of the amorphous content (I_{am}), the intensity at the minimum between the (110) peak and the overlapped (200) peak, using the formula:

$$\%CI = \frac{I_{200} - I_{am}}{I_{200}} * 100$$

Table S1 shows characterization data of functionalized wood veneers. After delignification, the relative lignin content reduced from ~20% to 3.45%. It gradually decreased further in TBirch and FBirch. The degree of crystallinity in the cell wall and cellulose content increased after delignification, as expected, and showed some additional changes in subsequent functionalizations

Table S1: Crystallinity index, solid densities, and carbohydrate analysis (relative contents) of functionalized wood samples.

Sample	% CI	Solid density (g/cm^3)	% Hemicellulose	% Cellulose	% Lignin
NBirch	46.2	1.4050	25.17	54.84	19.99
DBirch	53.6	1.4504	27.11	69.44	3.45
TBirch	52.5	1.4649	25.80	71.06	3.14
FBirch	55.1	1.4043	25.53	72.60	1.87

(TBirch, FBirch). The densities of DBirch and TBirch were higher due to wood densification during drying. FBirch has a similar density to NBirch but increased porosity due to lower fibril aggregation.

SAXS analysis: The interfibrillar distance was extracted using the equation, $d = 2\pi/q$, where d is interfibrillar distance and q is the peak center of Gaussian fit of the data in $I(q)*q^2$ vs q plot (see **Figure 4** in the main article) and are shown in **Table S2**.

Table S2: Interfibrillar distance in functionalized wood veneers.

Sample ID	Peak center of Gaussian fit (nm^{-1})	$d = 2\pi/q$
NBirch	1.62	3.88 nm
DBirch	1.83	3.43 nm
TBirch	2.03	3.09 nm
FBirch	1.54	4.07 nm

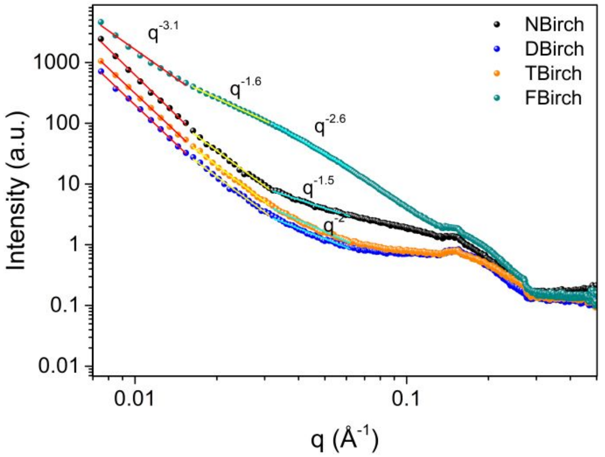

Figure S4: 1-D SAXS data (I vs q plot) of functionalized wood veneers.

Vibration frequencies: The active vibration frequencies of sensors were calculated by Fast Fourier Transformed analysis of the voltage signals and are plotted in **Figures S5** and **S6**. The dominant frequencies of all the sensors are around 17-18 Hz, which is close to the natural frequency of the cantilever beam (~17.9 Hz). The natural frequency of the cantilever beam was calculated using the following equation:[S2] $f = \frac{3.52}{2\pi L^2}\sqrt{\frac{EI}{\rho A}}$, where *I,* is a moment of inertia of the beam which was calculated using the equation, $I = \frac{bh^3}{12}$; E (195 GPa) and ρ (7850 kg/m^3) are Young's modulus and density of the beam material (stainless steel). L, b, and h are the length (150 mm), width (13 mm), and thickness (0.5 mm) of the beam.

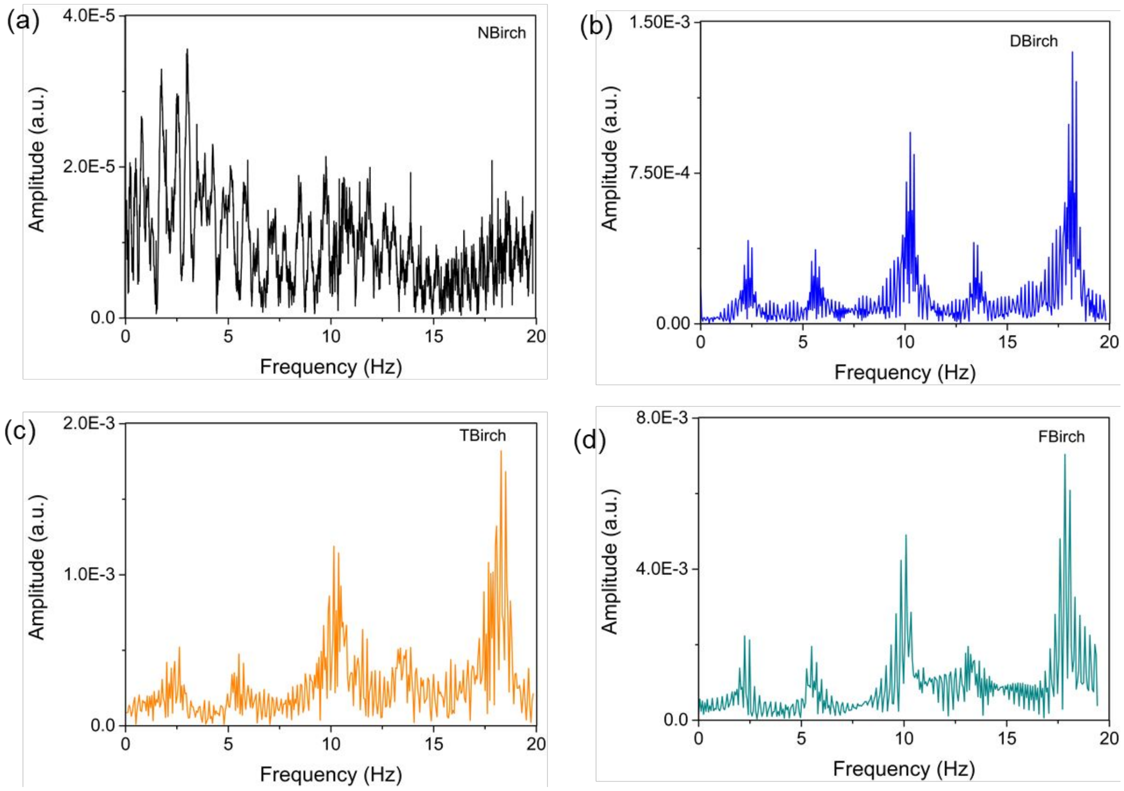

Figure S5: Frequencies of vibrations and sensing by NBirch, DBirch, TBirch, and FBirch (Fiber direction - 0°).

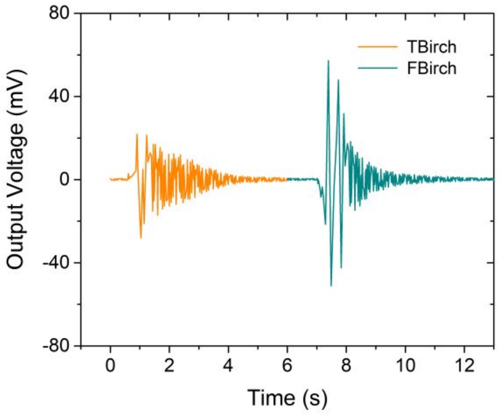

Figure S6: Piezoelectric output voltage generated by sensing of vibrations from the beam having the fiber orientation parallel to the beam length (fiber direction 0°) after prolonged storage of 5-6 months at ambient conditions (22 °C, RH≈53%).

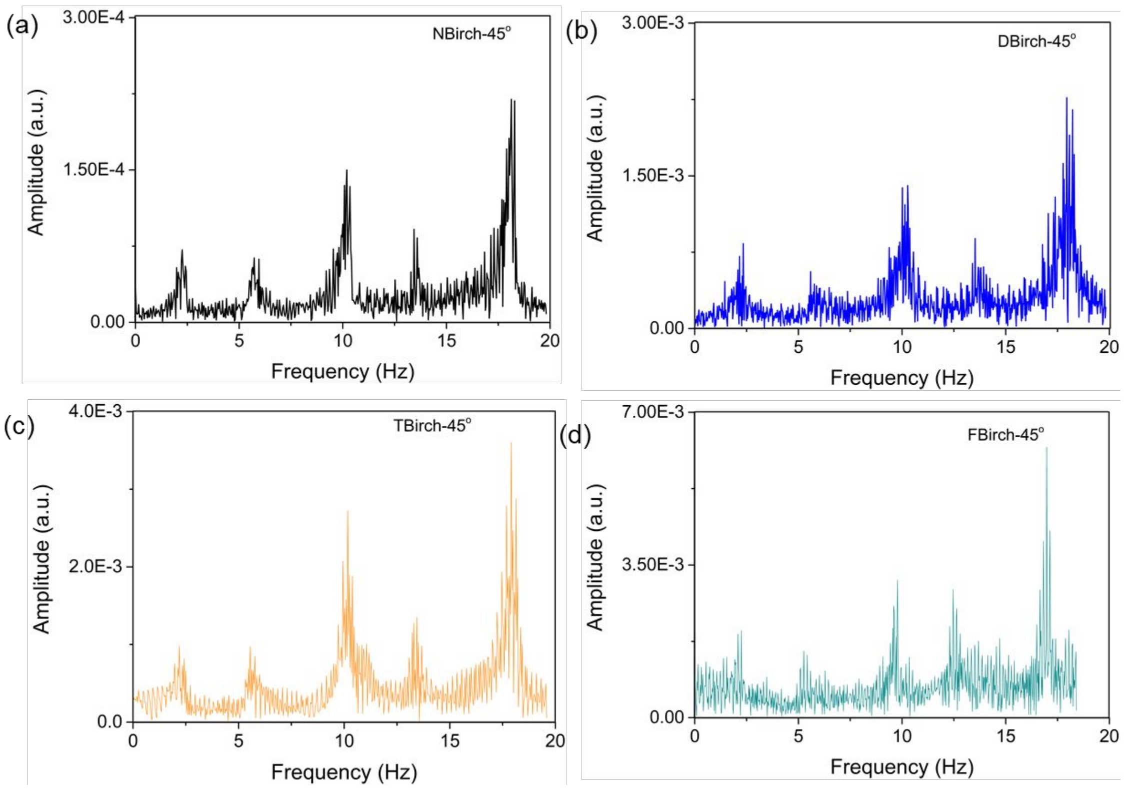

Figure S7: Frequencies of vibrations and sensing by NBirch, DBirch, TBirch, and FBirch (Fiber direction - 45°).

Triboelectric energy harvesting: The triboelectric nanogenerators are fabricated from the TBirch-Fbirch pair (each 1.3 x 2.5 cm^2). One side of the wood samples was first coated with silver paste and then attached with copper tape electrodes. Finally, a copper wire was soldered to copper electrodes. One part of the device was mounted on a linear motor shaft and the other one was mounted on a stopper. The two parts were brought into contact via a periodic motion of the linear motor with a frequency of 0.1-1 Hz (force ~25-30 N) and the output voltage was recorded using Keithley's DMM 7510.

Figure S8 shows the triboelectric energy harvesting performances of the TBirch and FBirch pair. Triboelectricity or contact electrification is based on the electron-donating and electron-accepting nature of the materials determined by their surface charge density. Various materials can be classified in a triboelectric series based on their electron-accepting (tribo-negative) or electron-donating (tribo-positive) capabilities. When two materials are in contact, one material will take the electron, and another will donate the electron based on their location in the triboelectric series. Wood is near zero in the triboelectric series. TEMPO-oxidation of delignified wood increases the electron-donating functionalities and wood becomes tribo-positive, whereas the presence of electron-withdrawing/accepting -CF_3 functionalities makes FBirch tribo-negative. A triboelectric nanogenerator fabricated from the TBirch-FBirch pair and its working mechanism is illustrated in **Figure S8a**. When periodically contacted, the electrons are donated by TBirch and accepted by FBirch, making TBirch contact-surface positive and FBirch contact-surface negative. Opposite charges are then generated under the electrodes to balance these surface charges and the electron flows from one material to the other and is measured using a voltage meter. **Figure S8b** shows the frequency-dependent output voltage. At 0.1 Hz contact frequency, the output voltage is around 4 V, and it increased to approximately 6 V at a contact frequency of 1 Hz. The high roughness of the fibrillar wood surface maximized the contact area and the extent of contact electrification so that higher output voltage was obtained. The charges generated during contact electrification eventually tend to neutralize. The charge recombination process is slower at higher frequencies, and the voltage output increases with increased frequency. Although the major contribution is from contact electrification, the piezoelectric properties of TBirch and FBirch also contribute to the overall output voltages. The robust wood materials ensure durability of the

triboelectric nanogenerator, and under these conditions, the output voltage is constant for at least 5000 cycles (**Figure S8c**).

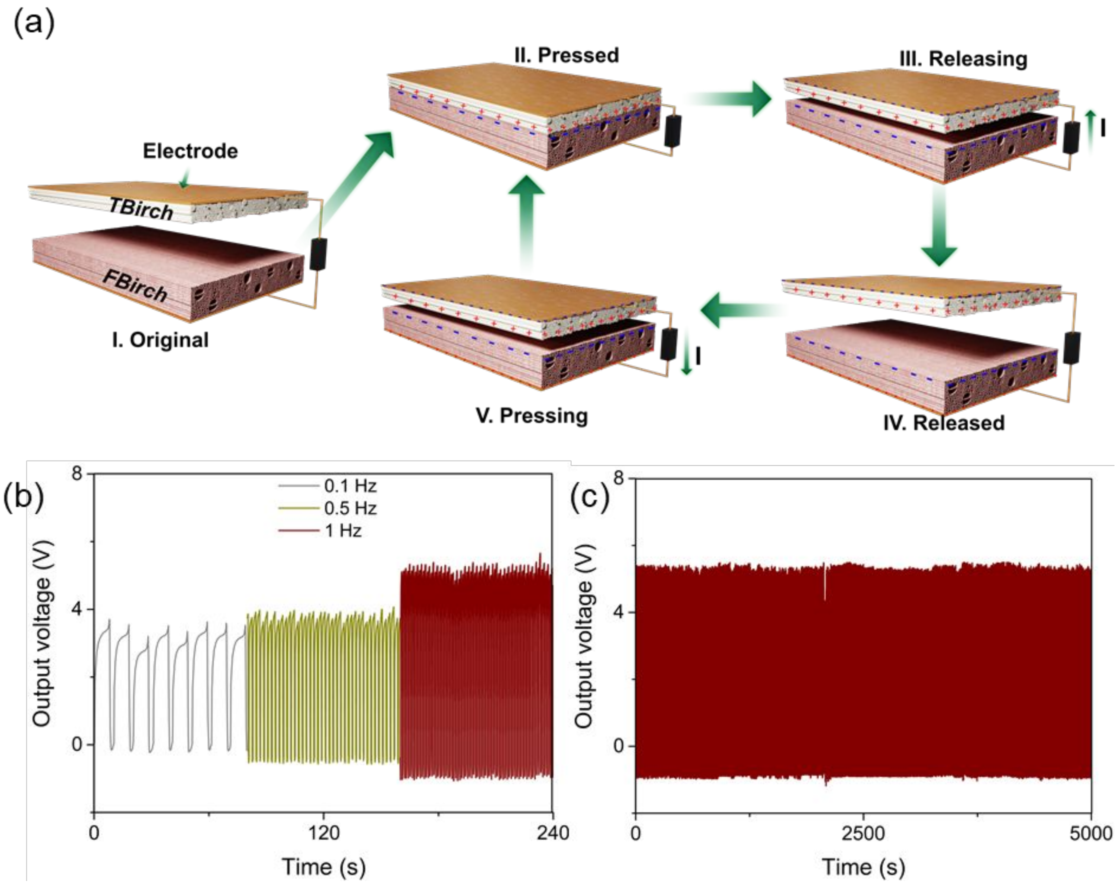

Figure S8: Triboelectric mechanical energy harvesting using TBirch and FBirch, (a) schematic of the working mechanism of TBirch-FBirch triboelectric nanogenerator (TENG). (b) Frequency dependent triboelectric voltage output from TBirch-FBirch TENG and (e) durability of the TENG for 5000 cycles at 1Hz.

Scheme S1: Overall procedure to prepare the functionalized wood materials.

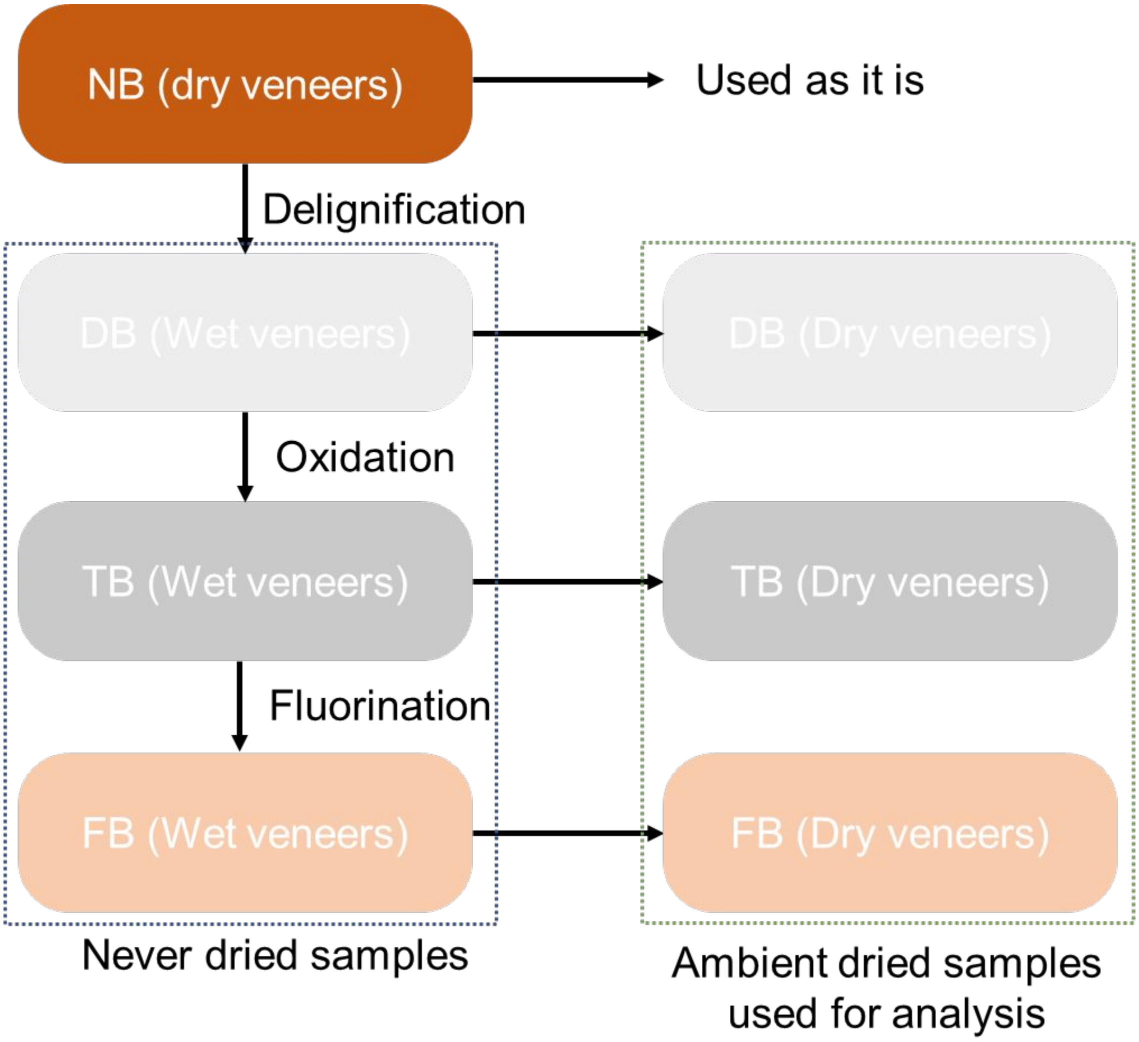

References

1. Segal, L. G.; Creely, J. J.; Martin Jr, A. E.; Conrad, C. M. An Empirical Method for Estimating the Degree of Crystallinity of Native Cellulose Using the X-Ray Diffractometer. *Text. Res. J.* **1959**, *29*, 786-794.

2. Yu, A.; Jiang, P.; Wang, Z. L. Nanogenerator as self-powered vibration sensor. *Nano Energy* **2012**, *1*, 418-423.
